# Impact of Plant Spacing and Nitrogen Rates on Growth Characteristics and Yield Attributes of Egyptian Cotton (*Gossypium barbadense* L.)

**DOI:** 10.3389/fpls.2022.916734

**Published:** 2022-05-12

**Authors:** Ibrahim A. E. Ibrahim, Waleed M. B. Yehia, Fouad H. Saleh, Sobhi F. Lamlom, Rehab Y. Ghareeb, Aly A. A. El-Banna, Nader R. Abdelsalam

**Affiliations:** ^1^Department of Plant Production, Faculty of Agriculture, Saba Basha, Alexandria University, Alexandria, Egypt; ^2^Agricultural Research Center, Cotton Research Institute, Giza, Egypt; ^3^Plant Protection and Biomolecular Diagnosis Department, Arid Lands Cultivation Research Institute, City of Scientific Research and Technological Applications, Alexandria, Egypt; ^4^Agricultural Botany Department, Faculty of Agriculture, Saba Basha, Alexandria University, Alexandria, Egypt

**Keywords:** cotton, growth characteristics, nitrogen, plant spacing, yield

## Abstract

This current study was performed to determine the influences of plant spacing, Nitrogen (N) fertilization rate and their effect, on growth traits, yield, and yield components of cotton (*Gossypium barbadense* L.) cv. Giza 97 during the 2019 and 2020 seasons. A split plot experiment in three replicates was utilized whereas the cotton seeds were planted at 20, 30, and 40 cm, as main plots and nitrogen at 75, 100, and 125%, was in subplots. The results revealed that the planting spacing at 40 cm significantly (*p* ≤ 0.01) increased plant height, number of fruiting branches per plant, number of bolls per plant, boll weight (BW), lint percentage (L%), seed cotton yield (SCY), lint cotton yield (LCY), seed index and lint index by 165.68 cm, 20.92, 23.93, 3.75 g, 42.01%, 4.24 ton/ha, 5.16 ton/ha, 12.05, 7.86, respectively, as average in both seasons. The application of N fertilizer rate at 125% caused a maximum increase in growth and yield parameters i.e., plant height (169.08 cm), number of vegetative branches (2.67), number of fruiting branches per plant (20.82), number bolls per fruiting branch (1.39), number of bolls per plant (23.73), boll weight (4.1 g), lint percent (41.9%), seed index (11.8 g), and lint index (8.2), while the plants treated with 100% N rates exhibited highest seed cotton yield (4.3 ton/ha) and lint cotton yield (5.6 ton/ha), as average in both seasons. Combining plant spacing at 40 cm between plants with a 100% N fertilizer rate recorded the highest lint cotton yield (5.67 ton/ha), while the highest seed cotton yield (4.43 and 4.50 ton/ha) was obtained from 125% N fertilizer rate under planting spacing 20 and 40 cm, respectively. Conclusively, a wide density (40 cm) with 125% N is a promising option for improved biomass, cotton growth, yield, physiological traits, and fiber quality.

## Introduction

Cotton (*Gossypium barbadense* L.) is a crucial cash crop in Egypt, providing fiber for textiles as well as edible oil (Gialvalis and Seagull, 2001; Ishaq et al., 2022; Zhao et al., 2022). During the 2019–2020 growing season, cotton was grown on 85,000 hectares with a total production of 250,000 bales. Cotton growth are significantly affected by climatic adversaries as well as seasonal management practices such as variety selection, sowing date, sowing method, plant spacing, water requirement, seed treatment and appropriate fertilizer application (Schaefer et al., 2018; Fahad et al., 2020; Zaman et al., 2021). Variety selection, sowing date, sowing method, plant spacing, water requirement, seed treatment, and appropriate fertilizer application are all important factors in cotton growth and development. It is important to plan improved management practices that enhance cotton yield potential. Cotton is extremely susceptible to abiotic stresses. Cotton growth and development are significantly influenced by climatic adversaries (Tung et al., 2018; Fahad et al., 2021d) and seasonal management practices (Fahad et al., 2021e) such as variety selection, sowing date, sowing method, plant spacing, water requirement, seed treatment, and appropriate fertilizer application (Muhammad et al., 2019; Fahad et al., 2021a,c).

It has been demonstrated that plant spacing is the most essential factor in enhancing the structures and increasing the cotton canopy’s photosynthetic potential (Bondada and Oosterhuis, 2001; Schaefer et al., 2018; Pabuayon et al., 2020; Fahad et al., 2021e; An et al., 2022), which is linked to cotton production strategy. Plant density has an effect on light absorption, moisture availability and wind movement, all of which have an impact on plant height, architecture, boll behavior, crop maturity and crop production (Khan et al., 2019; Fahad et al., 2021d). Reducing seeding rates may reduce input costs, maturity, fluff yield, and fiber quality may be negatively affected when the plant quantity is too low (Shah et al., 2021a). A further finding was that having a low plant density resulted in having a greater number of heavy bolls per plant, whereas having an increased plant density resulted in a drop in both the amount and weight of bolls (Bednarz et al., 2007). To better know the relationship between plant density and cotton productivity there are several research has been undertaken in this filed according to Bondada and Oosterhuis (2001) and Wei et al. (2022), several research has been undertaken. According to Chapepa et al. (2020) and Shah et al. (2021a), plant density increased in conjunction with the increase in LAI, increasing both yield and LAI. Poor management practices used throughout the blooming and boll formation stages have a negative impact on fiber quality parameters such as fiber strength, fiber fitness (or length), number of fibers/unit of length or uniformity index and fineness (Egelkraut et al., 2004; Main et al., 2014). Additional factors such as plant density and fertilizer have a significant impact on fiber quality (Boquet and Breitenbeck, 2000; Main et al., 2014).

High nitrogen requirements are a common limiting factor in crop growth based on their role in cotton photosynthesis and canopy development (Devkota et al., 2013; Muhammad et al., 2019; Gross, 2022; Rivero et al., 2022; Van Der Sluijs, 2022; Zhi et al., 2022). Because of this, it is the most crucial component in cotton fertilization to get a desirable yield (Bondada and Oosterhuis, 2001). Another study found that nitrogen fertilizer had a substantial effect on cotton growth, boll development, lint output and fiber quality (Devkota et al., 2013; Luo et al., 2018). As a result, nitrogen can improve salt tolerance and water productivity as well as nitrogen usage efficiency (Polychronaki et al., 2012; Devkota et al., 2013; Shah et al., 2017b, 2021a). The opposite is true: low nitrogen fertilization rates lead to sluggish growth and development, which in change results in low yield (Yang et al., 2011; Hafeez et al., 2019; Shah et al., 2021b). As a result, a number of studies have been done during recent decades to study the effect of N on cotton growth performance (Yang et al., 2011; Shah et al., 2017a; Chen et al., 2018). Many physiologically active molecules in cotton are affected by nitrogen fertilization (Huang et al., 2022; Javed et al., 2022). Chlorophyll, protein, enzymes, and phyto-hormones are just a few of the things that are affected by it (Dordas, 2009; Wang et al., 2022). Thus, nitrogen impacts cotton’s physiological features, which further impact on growth and morphological characters, which determines the final yield and quality (Dordas, 2009; Alitabar et al., 2012; Kumar et al., 2022). The bolls number, weight, and the quality of the fiber are all affected by nitrogen (Zhou et al., 2011; Khan et al., 2017a,b; Muhammad et al., 2019). According to preliminary findings, the top fruiting tillers were most vulnerable to N, which could explain why cotton reaches an early senescence stage earlier than expected (Liu et al., 2007; Tung et al., 2019; Fahad et al., 2021d). Therefore, this study was undertaken to define the optimal plant spacing and N rate for cotton development, physiology, and yield factors.

## Materials and Methods

### Experimental Field and Soil Analysis

**FIGURE 1:**
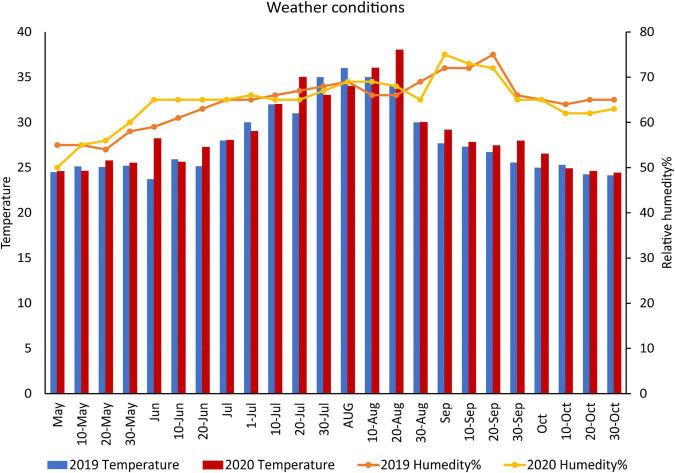
Weather conditions during the two growing seasons of cotton cultivation.

Field trials lasting 2 years took place at the Agricultural Research Center’s Sakha Station in Egypt. Through April and May of 2019 and 2020, cotton seeds (Cv. Giza 97) were sown in the fields. The climate of this area is characterized as hot summer, muggy, arid, and clear and the winters are cool, dry, windy, and mostly clear (Figure 1). To begin, soil physicochemical parameters have been studied in both summer and winter (Carter and Gregorich, 2007; see Table 1). To obtain soil samples, a 2.5 cm spiral auger was used to drill into each plot from two different depths (ranging from 0 to 25 cm) of soil. Each plot has three sub-samples taken from it to generate a composite sample for that plot. It was then ground into a fine powder to calculate the soil organic carbon (percent), N, P, and K available (mg.kg^–1^) from the samples, which were oven-dried at 40°C and crushed to fit through a 2 mm filter. E.C. was measured using established methods and soil pH was defined using the method of Carter and Gregorich (2007).

**TABLE 1:** The primary physiochemical properties of the experimental soil.

Year	PH	EC*	Organic matter%	Total N (mg/100 g)	CaCO_3_ (%)	Available P (mg/100 g)	Available K (mg/100 g)	Texture class
2019	7.8	1.34	1	35	4.8	1.4	30	Loam
2020	7.92	1.29	1.14	37	4.32	1.36	31	Loam

*EC, Soil electrical conductivity.

### Experimental Layout and Treatments

This current study was laid out under a split plot design using 3 replicates. The main plots were given planting spacing, whilst the sub-plots were given nitrogen fertilizer treatments. The experiment’s subplots were each 3.5 m long and 3 m broad (10.5 m^2^). Cottonseed was planted at three different planting space treatments: 20, 30, and 40 cm. The nitrogen fertilizers (Urea 46.5% nitrogen, Abu Qir Fertilizers Company) also consisted in three different rates of recommended dose as follows: 75% (253.125 kg N ha^–1^), 100% (337.5 kg N ha^–1^), and 125% (421.875 kg N ha^–1^) all nitrogen doses add in three times during plant growth. Before planting, the field received a base dosage of 150 kg P_2_O_5_ ha^–1^ and 225 kg K_2_O ha^–1^. Weeding, hoeing, insecticides and irrigation were all applied in a timely way to improve crop development.

### Data Recorded

#### Growth Parameters

Five plant sample were randomly taken from each plot to evaluate growth charters i.e., plant height (PH) (cm), Number of vegetative branches per plant (No V B/P), Number of fruiting branches per plant (No F B/P). The main stem number was noted at the first fruiting tiller arose was defined by designating the node immediately on top of the cotyledonary scores as number one and count the successive ascending nodes until the one that gave rise to the first fruiting branch.

#### Yield and Its Components

Seed cotton yield (ton/ha) and lint cotton yield (ton/ha) were determined by hand-harvesting 3 times from each treatment. The moisture level of the bolls was decreased to less than 11% after air drying, and seed cotton from 100 bolls was tested for boll weight during the first harvest. Using the total seed cotton output of 100 bolls as a starting point, the weight of a single boll was calculated. To calculate the proportion of lint in a 100-boll crop, divide the lint yield by the seed cotton weight of 100-bolls. The bolls number/fruiting branch (No B/FB) was computed by dividing the total number of open bolls into 10 plants by the total number of fruiting branches. The open bolls number per plant (No B/P) was calculated by calculating the number of open bolls on the 10 typical plants mentioned above before the first, second and third pickings in the first and second seasons. The average weight of 100-seeds in grams is known as the seed index. The lint index (LI) was determined using the formula:


Lintindex=(Seedindex×Lint%)/(100-Lint%)(Khan et al., 2010).

### Data Analysis

The analysis of variance (ANOVA) for all studied traits was performed utilizing the general linear model (GLM) procedure of the SAS 9.2 software for Windows (SAS Institute, 2011). Data were statistically evaluated using Fisher’s least significant difference (LSD) test at a 5%. Boxplots were done to show the difference in the application of plant space and nitrogen rates fertilization. Pearson correlation coefficients were used to access the associations among traits. In the R project (version 3.4.5), the ggplot2 package was used to draw a boxplot.

## Results

### Impact of Plant Space, Nitrogen Rates Fertilization and Their Interaction on Cotton Plant Development and Yield Parameters

Plant space, N fertilizer levels and their interaction were examined in Table 2 using an ANOVA. A significant (*P* > 0.01) relationship was found between plant space and nitrogen fertilizer rates, which was found to be related to plant height (PH), the first fruiting node (FFN), the vegetative branches number per plant (No VB/P, only in the second season), the fruiting branches number per plant (No FB/P, only in the first season), the number of bolls per fruiting N fertilizer rates did not affect the number of vegetative branches per plant in the second season (No VB/P) or on the first fruiting node (FFN, in both seasons). All growth and yield component parameters were shown to be significant impacted by the interaction among plant space and N fertilizer rate treatments in both seasons.

**TABLE 2:** ANOVA of the effects plant space, nitrogen rates and their interaction on growth, physiological and yield parameters of cotton plants.

Source of variance	Plant height		First fruiting node		No. of vegetative branches/plant		No. of fruiting branches/plant		No. bolls/fruiting branch		No. of bolls/plant	
	2019	2020	2019	2020	2019	2020	2019	2020	2019	2020	2019	2020
Plant spacing (P)	***	***	***	**	ns	**	***	ns	***	ns	***	***
N fertilizer (N)	***	***	ns	ns	**	ns	**	**	***	***	***	***
P × N	***	***	**	***	**	***	**	***	**	***	***	***
CV	1.05	0.91	3.02	3.48	16.42	19.62	4.29	4.92	7.66	7.68	5.12	4.29
*R* ^2^	0.96	0.97	0.75	0.74	0.69	0.74	0.65	0.82	0.85	0.89	0.91	0.95
RMSE	1.71	1.49	0.22	0.25	0.42	0.42	0.87	0.99	0.092	0.090	1.41	0.93
	**Boll weight**		**Lint cotton**		**Seed cotton yield**		**Lint cotton yield**		**Seed index**		**Lint index**	
Plant spacing (P)	***	***	***	***	**	**	**	**	***	***	***	***
N fertilizer (N)	***	***	***	***	***	***	**	***	***	***	***	***
P × N	***	***	***	***	***	***	**	***	***	***	***	***
CV	5.25	3.64	1.49	1.33	2.41	5.87	3.58	4.98	2.28	3.19	2.35	2.57
*R* ^2^	0.94	0.96	0.89	0.92	0.91	0.77	0.93	0.90	0.96	0.93	0.97	0.97
RMSE	0.18	0.13	0.61	0.55	0.27	0.62	0.50	0.61	0.25	0.35	0.17	0.19

ns, **, *** indicate not significant, significant at 5% (p ≤ 0.05), significant at 1% (p ≤ 0.01) and significant at 0.1% (p ≤ 0.001) probability level, respectively.

*CV, coefficient of variation; RMSE, root mean square error; R^2^, coefficient of determination.*

### The Impacts of Plant Space and Nitrogen Levels on Growth and Physiological Parameters of Cotton Plants

During the 2019 and 2020 seasons, plant space and N fertilizer treatment rate showed a significant impact on the morphological characters of cotton plants. As presented in Table 3, planting at 40 cm showed the maximum plant height (165.67 and 165.69 cm) in both seasons, respectively. Whereas application of 125% N fertilizer exhibited the tallest plants (169.02 and 169.14 cm) in 2019 and 2020, respectively. In the case of the FFN, the results showed that planting at 40 cm was the best and earlier for the mean performance values (7.31 and 7.26 node) in the 2019 and 2020 seasons. Nitrogen fertilizer did not affect significantly on this trait. According to the results in Table 3, the No. VB/P didn’t affect significantly by planting space in the first season, but in the next season, planting at 20 and 40 cm recorded the highest No. VB/P (2.31 and 2.38), respectively. Whereas application of 125%N fertilizer recorded the highest No. VB/P in the first season, meanwhile, in the next season, the results showed a non-significant effect due to the nitrogen fertilizer rates. Regard, the No FB/P. The maximum No. F.B./P (21.41) was recorded under planting at 40 cm in 2019, while there was no significant difference between planting spacing in the 2020 season. The increase in nitrogen application rate influenced the No FB/P. The highest No FB/P (20.72 and 20.93) were observed under125% N fertilizer in both seasons, respectively. The results demonstrated that in 2019, plant spacing have a substantial influence on No B/FB. Planting at 40 showed the highest No. B/FB (2.30), while plant space displayed an insignificant difference in the 2020 season. Referring to the nitrogen fertilizer rates in 2019 and 2020 seasons, 125% N fertilizer was the best treatment with relevance to No B/FB (1.38 and 1.41), respectively. In the case of No B/P, the plants are sown at 40cm showed the highest No. B./P (24.52 and 23.35) during both growing seasons followed by 20 and 30 cm. Nitrogen rates also significantly influenced this trait, the highest mean values of the No B/P (23.61 and 23.85) were observed under 125% nitrogen fertilizer rate, as shown in Table 3.

**TABLE 3:** Impacts of plant space and N fertilization level on some physiological constraints of cotton combined through 2 years (2019–2020).

Treatments	Plant height (cm)		First fruiting node		No. of vegetative branches		No. of fruiting branches/plant		No. bolls/fruiting branch		No. of bolls/plant	
	2019	2020	2019	2020	2019	2020	2019	2020	2019	2020	2019	2020
**Plant space (P)**												
20 cm	162.16 b	161.34 b	7.71 a	7.63 a	2.63 a	2.31 a	19.61 b	20.04 a	1.10 c	1.61 a	21.70 b	21.97 b
30 cm	162.54 b	161.74 b	7.48 ab	7.45 ab	2.70 a	1.77 b	19.86 b	20.14 a	1.21 ab	1.55 a	20.88 b	19.80 b
40 cm	165.67 a	165.69 a	7.31 b	7.26 b	2.36 a	2.38 a	21.41 a	20.43 a	1.30 a	1.26 a	24.52 a	23.35 a
LSD_0_._05_	2.75	2.25	0.28	0.25	ns	0.48	1.32	ns	0.13	ns	1.79	0.53
**N Fertilization (N)**												
75%	156.93 c	156.07 c	7.39 a	7.54 a	2.22 b	2.01 a	19.81 b	19.78 b	1.04 c	0.98 c	20.75 b	19.17 b
100%	163.43 b	163.55 b	7.52 a	7.43a	2.49 b	2.11 a	20.35 ab	20.01 ab	1.20 b	1.13 b	22.74 a	23.10 a
125%	169.02 a	169.14 a	7.62 a	7.38 a	2.99 a	2.35 a	20.72 a	20.93 a	1.38 a	1.41 a	23.61a	23.85 a
LSD_0_._05_	1.75	1.53	ns	ns	0.46	ns	0.84	0.99	0.091	0.094	1.17	0.95
**Interaction**												
P × N	**	**	**	**	**	**	**	**	**	**	**	**

** indicate significant at 1% probability level. Different lowercase letters indicate statistically significant differences between treatments (p ≤ 0.05), as performed by the least significant difference (Fisher’s LSD) test.

### The Effects of Plant Space and Nitrogen Rates on Yield and Yield Components

Boll weight (BW), lint percentage (L%), seed cotton yield (SCY/ha), lint cotton yield (LCY/ha), seed index (SI) and lint index (LI) of cotton were significantly impacted by plant space and N fertilizer rates in both years (Table 4). Plant spacing significantly affected BW, whereas 40 cm between cotton plants gave the highest BW of (3.73 and 3.78 g) in the 2019 and 2020 seasons, respectively. Additionally, nitrogen fertilizer rates application varied significantly *(p* ≤ *0.001)*. The application of 125% nitrogen fertilizer rate exhibited the heaviest BW (4.20 and 4.07 g) in both seasons, respectively. The lint percentage was affected by plant space and affected by the N fertilizer rate. Planting at 40 and 30 cm recorded the highest lint percentage (42.13 and 42.32%) in both seasons, in respect. Increasing the N fertilizer levels from75 to 125% improved the lint percentage and observed the highest percentage compared to the low N fertilizer rate (Table 4). During both years, 40 cm plant space yielded the highest SCY (4.29, and 4.19 ton/ha^–1^) and LCY (5.43 and 4.89 ton/ha^–1^) in both season, respectively. Nitrogen rats had a significant impact on SCY and LCY. The application of 100% nitrogen rate indicated the greatest SCY in the first season of (4.44 ton/ha^–1^), while125% nitrogen fertilizer rate showed the highest SCY of (4.26 ton/ha^–1)^ in the second season. Concerning, the LCY, 100% nitrogen application observed the highest LCY of (5.97 and 5.23 ton/ha^–1^) in both seasons, respectively. The seed index and lint index increased with increasing plant space. The highest seed index (12.14 and 11.97) was produced by space 40 cm between cotton plants in the first and second season, respectively, regarding lint index also 40 cm between cotton plants produced the highest mean value (8.16 and 7.57) in 2019 and 2020, respectively, while application of 125%of N fertilizer rate recorded the highest seed index and lint index in both seasons.

**TABLE 4:** Impacts of plant space and N fertilization level on yield, and yield components through 2 years (2019–2020).

Treatments	Boll weight (g)		Lint cotton (%)		Seed cotton yield (ton/ha)		Lint cotton yield (ton/ha)		Seed index		Lint index	
	2019	2020	2019	2020	2019	2020	2019	2020	2019	2020	2019	2020
**Plant space (P)**												
20 cm	3.41 b	3.50 b	40.26 b	39.90 b	4.05 c	3.91 b	5.17 b	4.45 b	10.56 c	10.32 c	7.10 c	7.43 a
30 cm	3.64 ab	3.77 a	41.81 a	42.32 a	4.09 b	4.01 b	5.13 b	4.68 ab	11.16 b	11.17 b	7.62 b	7.22 b
40 cm	3.73 a	3.78 a	42.13 a	41.90 a	4.29 a	4.19 a	5.43 a	4.89 a	12.14 a	11.97 a	8.16 a	7.57 a
LSD_0_._05_	0.299	0.26	0.58	0.68	0.18	0.16	0.25	0.36	0.13	0.25	0.23	0.15
**N fertilization (N)**												
75%	3.12 c	3.35 c	40.39 b	40.41 b	4.05 c	3.74 c	5.30 b	4.64 b	10.58 c	10.37 c	6.77 b	6.40 c
100%	3.46 b	3.64 b	41.82 a	41.83 a	4.44 a	4.01 b	5.97 a	5.23 a	11.27 b	11.33 b	8.022 a	7.45 b
125%	4.20 a	4.07 a	42.00 a	41.87 a	4.10 b	4.26 a	4.73 c	4.14 c	12.02 a	11.76 a	8.08 a	8.38 a
LSD_0_._05_	0.199	0.13	0.63	0.57	0.08	0.21	0.19	0.23	0.26	0.36	0.18	0.21
**Interaction**												
P × N	**	**	**	**	**	**	**	**	**	**	**	**

*Different lowercase letters indicate statistically significant differences between treatments (p ≤ 0.05), as performed by the least significant difference (Fisher’s LSD) test.*

### Interaction Between Cotton Planting Space and Nitrogen Rates Treatments Space

**FIGURE 2:**
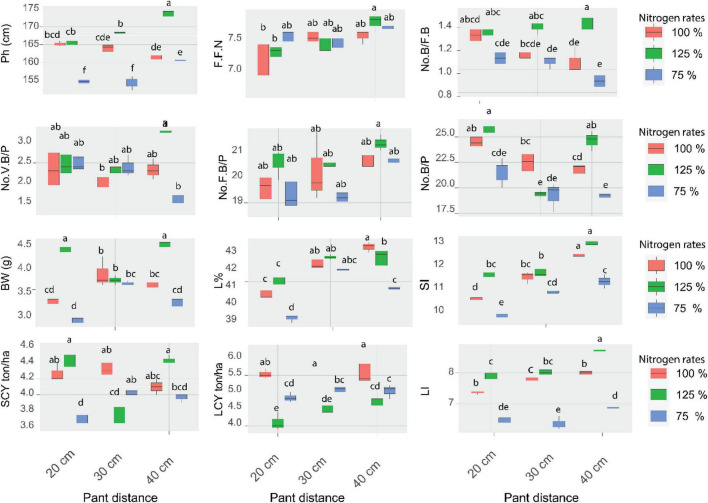
The effects of a combination of varied plant spacing (20, 30, and 40 cm) and nitrogen rates (75, 100, and 175 percent) on 12 cotton attributes found in field trials were integrated using data from the 2019 and 2020 seasons. The least significant difference (Fisher’s LSD) test shows that different lowercase letters on error bars indicate statistically significant differences between treatments (*p* 0.05).

The effect of planting space and nitrogen rates were significant for the plant height as shown in Figure 2. The plant height significantly varied for the interactive effect of spacing and nitrogen rates application. The tallest plant (173.5 cm) was observed underplant space of 40 cm with 125% N fertilizer rate, while the shortest plant (154 cm) was recorded under 20 and 30 cm with 75% Nfertilizer rate in both seasons. Also, for the FFN the results showed that the 40 cm space with 125% N fertilizer was the best and the earlier plants with a value of (7.76 nodes) in both seasons. BN was significantly increased by planting space and nitrogen fertilizer interaction. 40 cm space between cotton plants in combined with 125% N fertilizer exhibited the highest number of No. VB/P of (2.326) and No. of FB/P of (21.36) in the first and second season. Whereas, the lowest No. VB/P was observed at 40 cm with nitrogen rates of 75%. Regarding No. FB/P, planting at 30 cm with a nitrogen rate of 75% recorded the lowest value, also planting at 40cm with 125% N recorded the highest No. B/FB (1.40). Planting the cotton seed at 40 cm between hills with 100 and 125% N fertilizer resulted in the highest seed index of (12.26 and 12.76), respectively. while planting at 20 cm with 75% N fertilizer exhibited the lowest seed index (9.60) in both seasons. The interaction between plant spacing and nitrogen rates had also a remarkable impact on the No.B/P in both growing seasons (Figure 2). The maximum No. B/P (25.70) was observed at 20 cm with nitrogen rates of 125% during the 1st and 2nd growing seasons. The bolls number and weight were impacted by plant space with N fertilizer rate interaction (Figure 2). The maximum BW was recorded at planting space 20 or 40 cm in companied with 125% nitrogen fertilizer application rate. Whereas the highest No. B/P was observed from planting at 20 cm with 125% nitrogen fertilizer followed by 40 cm and 125% nitrogen rate. The interactive influence of planting space and nitrogen rates resulted in substantial variations in L%. The maximum L% (43.03%) was observed in the plants space of 40 cm with nitrogen rates of 100% during both growing seasons. Seed index was affected by interactions of plant space and N rate (Figure 2), increased plant space improved seed index (Figure 2). The results showed that the 40 cm space with100 and 125% nitrogen recorded the best seed index value of (12.26 and 12.76) in both seasons, respectively. The interactive influence of plant space and nitrogen rates resulted in substantial variations in the LI. The maximum LI was observed at a space of 40 cm with nitrogen rates of 125% during both growing seasons. The interactive influence of planting space and nitrogen rates resulted in substantial variations in the SCY. The highest mean value of SCY (4.43 ton/ha) was observed at 20 and 40 cm with 125% N fertilizer rate during the 1st and 2nd growing season followed by 30 and 20cm with 100% N fertilizer rate. LCY showed significantly affected to N fertilizer rates, plant space and their interaction (Figure 2). Planting cotton at 40 and 30 cm in combined with 100% nitrogen fertilizer exhibited the highest mean value of LCY (5.67 and 5.66 ton/ha), respectively. The results suggested that LCY could be increased through coordination of N fertilizer rate and plant space, for instance moderate N fertilizer rate at any plant space.

### Correlation Between Studied Traits

**FIGURE 3:**
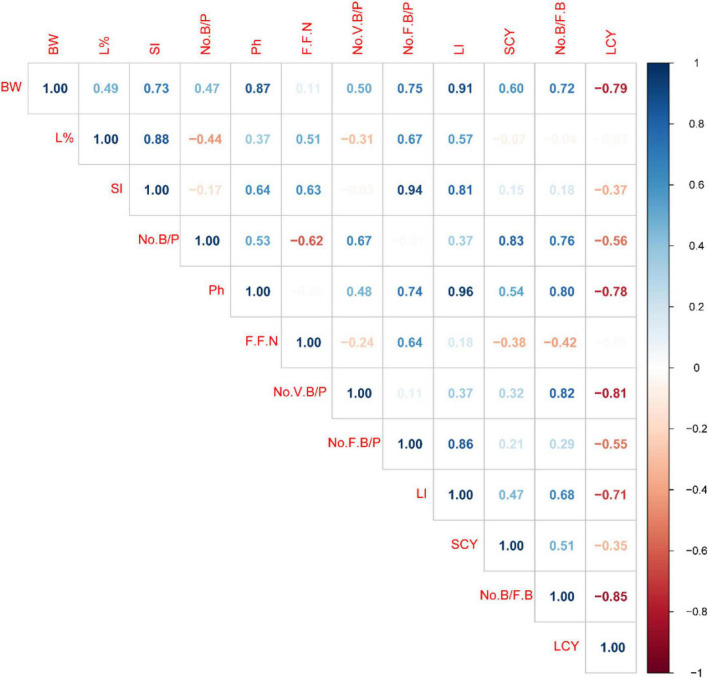
Pearson’s correlation coefficients for 12 attributes tested under various plant spacing and nitrogen treatment rates (Combined analysis of two successive seasons of 2019 and 2020). SCY, Seed cotton yield; LCY, Lint cotton yield; SI, Seed index; LI, Lint index; BW, Boll weight; L%, Lint percentage; No. VB/P, Number of vegetative branches; No. B/FB, Number of bolls per fruiting branch; No. FB/P, Number of fruiting branches per plant; No. B/p, Number of bolls per plant Positive correlation is shown by blue, while negative correlation is indicated by red.

Positive and negative correlation were recorded between the studied morphological and yield traits (Figure 3). The correlation among boll weight, lint percentage, No B/P, plant height, No.V B/P, No. F B/P, lint index, SCY, No. B/FB and seed index was significantly positive at both plant space and nitrogen rates. The PH exhibited a positive relationship with No. VB/P, No. FB/P, lint index, SCY and No. B/FB at both levels, however it had a significantly negative correlation with LCY. Likewise, FFN expressed a positive correlation with No. F B/P, while it demonstrated a negative relationship with No. V B./P, SCY and No. B/FB. The association between No. VB/P, lint index and No. B/FB were significantly positive. However, No. V B/P exhibited a significantly negative correlation with (LCY). Similarly, the relationship between No. FB/P and lint index was strongly positive. It is important to understand the correlation among yield attributes that directly contribute to enhanced cotton productivity. The SCY indicated a positive and highly significant association with BW, No. B/P, PH and lint index. The direct selection of these attributes may improve the selection efficiency of yield in cotton.

### Interrelationship Between Combinations of Plant Spacing and Nitrogen Rates Application (Based on Yield and Growth Parameters)

**FIGURE 4:**
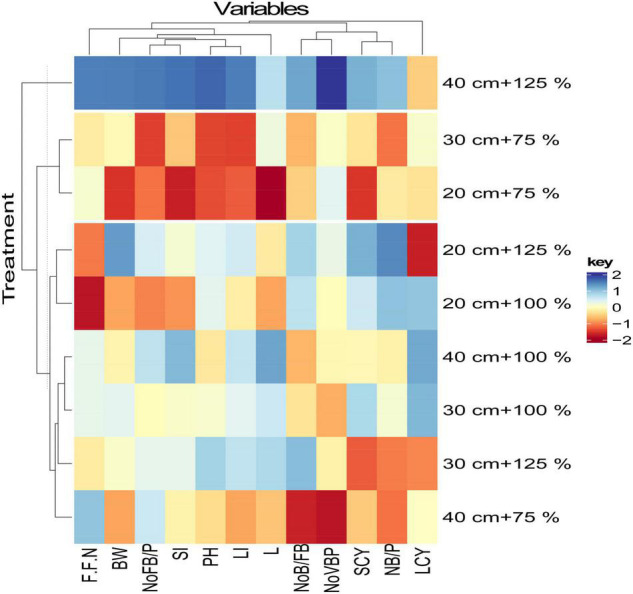
Clustering analysis presents the relationships between plant space, nitrogen fertilization treatment and studied traits. In the ballots, the hierarchical clustering analysis with the Euclidean space using the principal component scores and Ward’s technique as the process of linkage was used. L%, lint percentage; FFN, first fruiting node position; No. F B/P, number of fruiting branch per plant; SI, seed index; SCY, seed cotton yield; No. B/P, number of boll per plant; L.I., lint index; PH, plant height, boll weight; No. V B/P, number of vegetative branch per plant; No. B/FB, number of bolls per fruiting branch; LCY, lint cotton yield.

The hierarchical clustering clearly distinguished the interrelationship between combinations of plant space and nitrogen rates application (7 combinations) according to their performance of yield and growth parameters (Figure 4). As regards the relationship between plant space and nitrogen rates treatments, two main clusters were characterized. The first cluster was formed by the combination’s treatment of A (40 cm + 125% N), in this group, treatment A provided the highest values for the majority of traits, except for LCY. The second cluster is divided into two subclusters, the first subclusters was formed by the combination of B (40 cm + 100% *N*), C (30 cm + 100% *N*), and D (40 cm + 75% *N*), whereas the treatments B and C, were the closest sub-clusters. For treatment B and C, showed the highest positive effects on LCY, indicating the best parameters under such plant space and nitrogen rates application. The treatment D negatively affected a majority of studied traits except for the first fruiting node position followed by the No.FB/P. The second sub-cluster included each of the treatment E (30 cm + 125% *N*), F (20 cm + 75% *N*), and G (30 cm + 75% *N*), whereas the treatments F and G were the closest sub-clusters. Overall, the combinations of fertilization treatments in the second sub-cluster (including E, F, and G subclusters) showed an opposite pattern with the treatments combinations of the first cluster, as all studied traits were negatively affected showing lower overall performance, especially for the G treatment which indicated the lowest value for all measured parameters.

## Discussion

Several investigations have been performed to investigate the impacts of plant spaces (Bednarz et al., 2007; Hafeez et al., 2018; Fahad et al., 2021b) and N fertilization rate (Boquet and Breitenbeck, 2000; Bondada and Oosterhuis, 2001) in terms of cotton yield and its yield components. Others have observed interactions between plant space and N fertilization (Ali et al., 2007; Zaman et al., 2021; Van Der Sluijs, 2022; Zhi et al., 2022). The impacts of plant space and N rates were explored in the current study, with an emphasis on their interaction. Cotton yield rose with wider plant space and N rates application, which can be explained by an increase in growth and yield components. The increased No.VB/P, boll numbers, plant height, branch number, boll weight, cotton yield, seed index and lint index were due to the wider plant space (Figure 2 and Tables 2, 3). These findings are related to those of Rinehardt et al. (2004) and Rafi et al. (2015), they indicated that plant height was found to be significantly influenced by plant space, as plants luxuriously utilized all resources and light interception was also better. Plant space affects light interception, moisture availability, nutrient uptake, humidity, and weed infestation (Stephenson, IV et al., 2011; Zhanbota et al., 2022) and thus influence plant height, fruiting behavior, maturity, and final yield. More competition among plants suppresses plant growth under narrow spacing. A wider plant space resulted in a shorter internodal space (Alfaqeih et al., 2002; Emaish et al., 2021). This is in affirmation with the earlier findings of Stephenson, IV et al. (2011), who concluded that higher plant density decreased the number of monopodial and sympodial branches. With the increase in plant spacing, the number of sympodial branches per plant also increased. Also, Alfaqeih et al. (2002) also reported similar results. An increase in the number of fruit branches per plant in low planting density could be due to less competition and more space available for the growth of plants. The number of plants per area was greater in narrow spacing treatments. The plants in the narrow spacing (20 cm) were dense (71,428 plants ha^–1^), while at wider spacing (40 cm) the number of plants was lower, i.e., 35,714 plants ha^–1^. Similar findings were reported by Delaney et al. (2002), Singh et al. (2012), and Brodrick et al. (2013). By increasing spacing, it was observed that boll weight increased, which led to the highest seed cotton yield. Boll weight showed a decreasing trend with the decrease in plant space as well as low nitrogen rates. Heavier bolls in wider spacing may be because of less competition amongst crop plants, resulting in efficient consumption of all resources (Table 3). These findings are found to be similar to Alfaqeih et al. (2002), Ali et al. (2007), Rafi et al. (2015), Alsalem et al. (2022), and Zhi et al. (2022). They reported that wider space increased the number of branches per plant and boll weight which was due to less competition between plants. The results were similar to those reported by Alfaqeih et al. (2002) and Morsy et al. (2022) they reported an increase in the number of bolls per plant was a direct consequence of more fruit branches per plant. In addition, Iqbal and Khan (2010) and Hashem et al. (2022) revealed that an increase in the number of bolls per plant with an increase in plant space can reduce competition between plants. Results also showed that crop sowed with 40 cm plant spacing significantly (*P* < 0.01) produced the highest seed and lint indexes. The abundance of space would have allowed the plants to absorb more water and nutrients, resulting in a higher number of sympodial branches. This would have resulted in more bolls per plant in the end. Furthermore, the maximum number of bolls may be attributable to improved photosynthate assimilation and translocation. These findings were similar to Sisodia and Khamparia (2007) and Ahmed et al. (2021) they stated the plant grows taller with respect to vertical space and produces a greater leaves number and sympodial branches per plant.

The use of optimum N fertilizer improves a variety of physiological and metabolic activities, including photosynthesis and nitrogen metabolism, which is a critical reducing factor in high cotton productivity and quality. As a result, one of the most essential ways to boost cotton output is to apply N fertilizer (Boquet and Breitenbeck, 2000; Muhammad et al., 2019; Yousaf et al., 2021; Ghareeb et al., 2022). Many research has demonstrated that a sufficient amount of N nutrition may boost cotton dry matter and growth rate at all stages (Luo et al., 2009; Fouda et al., 2020a). Furthermore, it enhances the dry matter distribution ratio (Luo et al., 2018; El-Naggar et al., 2020; Elkobrosy et al., 2022), enhances photosynthetic product accumulation and transport (Liu et al., 2015; Fouda et al., 2020b) and promotes production (Clawson et al., 2006; Ahmed et al., 2021). The nitrogen application of 100 and 125% increased the lint percentage, boll weight, lint cotton yield, seed cotton yield, seed index, lint index, plant height, vegetative branches number, bolls number per fruiting branch, fruiting branches number per plant, bolls number per plant in cotton compared to where low nitrogen rate was applied (Figure 2). In line with earlier research of Dong et al. (2012), Wang et al. (2016), Abdelsalam et al. (2019c), Abualnaja et al. (2021a,b), and Ahmad et al. (2022), *an* increased N rate (N0–N2) boosted yield and boll weight substantially. The results showed that enhanced yield was linked to increased boll weight and nitrogen plays a significant role in the production of boll weight and is the key component influencing yield. Our findings revealed that when a 125% N rate was applied, morphological and yield traits increased as compared to when a low nitrogen rate was applied (Figure 2). The use of nitrogen has been shown to boost plant height in a variety of crops (Kumbhar et al., 2008; Dong et al., 2010; Fahad et al., 2021d; Abbas et al., 2022; Abdelsalam et al., 2022). Our results were in line with (Kumbhar et al., 2008; Abdelsalam et al., 2019a), who indicated that nitrogen has a role in the *plant* rapid vegetative development and nitrogen deficiency influences the growth and yield of seed cotton. The goal of better management is to maximize N fertilizer (Rinehardt et al., 2004; Abdelsalam et al., 2019b; Zhao et al., 2019, 2021). The results of our study show that nitrogen fertilization has a significant influence on the number of bolls generated per plant (Table 2). This might be attributed to nitrogen fertilizer because the cotton plant is particularly susceptible to nitrogen absorption. These outcomes are comparable to Rashidi and Seilsepour (2011) they reported that because cotton is more sensitive to nitrogen than other crop plants, an increase in nitrogen increases the bolls number per plant significantly.

## Conclusion

According to the findings of this research, sowing density and nitrogen fertilization had a significant impact on the development and physiology of the cotton crop. When comparing low density plants to high density crops, it was found that the accumulation of reproductive structure biomass was greater throughout the peak bloom, boll set and maturity stages of the crop. It was increased nitrogen intake at various developmental stages that resulted in the increase in reproductive organ biomass creation under low density. Planting density had little effect on the buildup of reproductive organ biomass during the early reproductive phase, but it had a considerable effect on the filling of the bolls later in the reproductive phase. Crops with a low or moderate density generated fiber with a better grade than crops with a high density. In conclusion, low density (40 cm) with 125% nitrogen fertilizer is a favorable management approach in terms of enhanced biomass production, nutrient absorption and yield compared to other management strategies space.

## Data Availability Statement

The raw data supporting the conclusions of this article will be made available by the authors, without undue reservation.

## Author Contributions

II, WY, FS, and AE-B: data curation, formal analysis, funding acquisition, methodology, and resources. II, WY, FS, AE-B, SL, RG, and NA: writing—original draft. SL and NA: writing—review and editing. All authors contributed to the article and approved the submitted version.

## Conflict of Interest

The authors declare that the research was conducted in the absence of any commercial or financial relationships that could be construed as a potential conflict of interest.

## Publisher’s Note

All claims expressed in this article are solely those of the authors and do not necessarily represent those of their affiliated organizations, or those of the publisher, the editors and the reviewers. Any product that may be evaluated in this article, or claim that may be made by its manufacturer, is not guaranteed or endorsed by the publisher.
